# Metformin and small for gestational age babies: findings of a randomised placebo-controlled clinical trial of metformin in gestational diabetes (EMERGE)

**DOI:** 10.1007/s00125-024-06252-y

**Published:** 2024-08-31

**Authors:** Fidelma Dunne, Christine Newman, Alberto Alvarez-Iglesias, Paula O’Shea, Declan Devane, Paddy Gillespie, Aoife Egan, Martin O’Donnell, Andrew Smyth

**Affiliations:** 1 College of Medicine, Nursing and Health Sciences, University of Galway, Galway, Ireland; 2 HRB Clinical Research Facility Galway, University of Galway, Galway, Ireland; 3 Galway University Hospital, Newcastle Road, Galway, Ireland; 4 School of Nursing and Midwifery, University of Galway, Galway, Ireland; 5 School of Business and Economics, University of Galway, Galway, Ireland; 6 Mayo Clinic, Rochester, MN USA

**Keywords:** Gestational diabetes, Metformin, Small for gestational age

## Abstract

**Aims/hypothesis:**

Gestational diabetes mellitus (GDM) is associated with adverse perinatal outcomes because of suboptimal glucose management and glucose control and excessive weight gain. Metformin can offset these factors but is associated with small for gestational age (SGA) infants. We sought to identify risk factors for SGA infants, including the effect of metformin exposure on SGA status.

**Methods:**

In this prespecified secondary analysis of the EMERGE trial, which evaluated the effectiveness of metformin vs placebo in treating GDM and found reduced gestational weight gain and longer time to insulin initiation with metformin use, we included women with a live-born infant and known infant birthweight and gestational age at delivery. We compared the numbers of SGA infants in both groups and explored baseline predictive factors to help identify those at highest risk of delivering an SGA infant.

**Results:**

Baseline maternal characteristics were similar between SGA and non-SGA pregnancies. On multivariable-adjusted regression, no baseline maternal variables were associated with SGA status. Mothers of SGA infants were more likely to develop pre-eclampsia or gestational hypertension (18.2% vs 2.0%, *p*=0.001; 22.7% vs 5.4%, *p*=0.005, respectively); after multivariable adjustment, pre-eclampsia was positively associated with SGA status). Among SGA pregnancies, important perinatal outcomes including preterm birth, Caesarean delivery and neonatal care unit admission did not differ between the metformin and placebo groups (20.0% vs 14.3%, *p*=1.00; 50.0% vs 28.6%, *p*=0.25; 13.3% vs 42.9%, *p*=0.27, respectively).

**Conclusions/interpretation:**

Pre-eclampsia was strongly associated with SGA infants. Metformin-exposed SGA infants did not display a more severe SGA phenotype than infants treated with placebo.

**Trial registration:**

Clinical Trials.gov NCT02980276; EudraCT number: 2016-001644-19

**Graphical Abstract:**

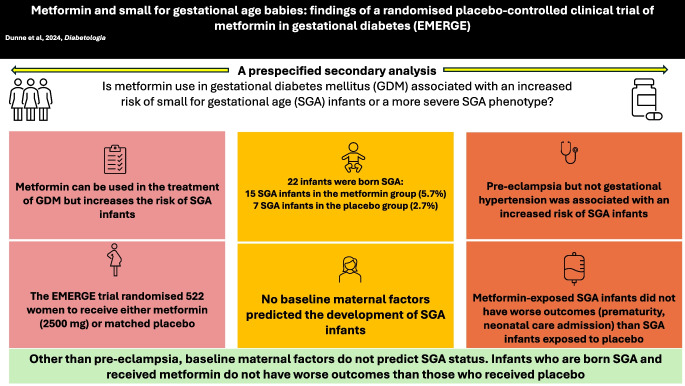

**Supplementary Information:**

The online version of this article (10.1007/s00125-024-06252-y) contains peer-reviewed but unedited supplementary material.


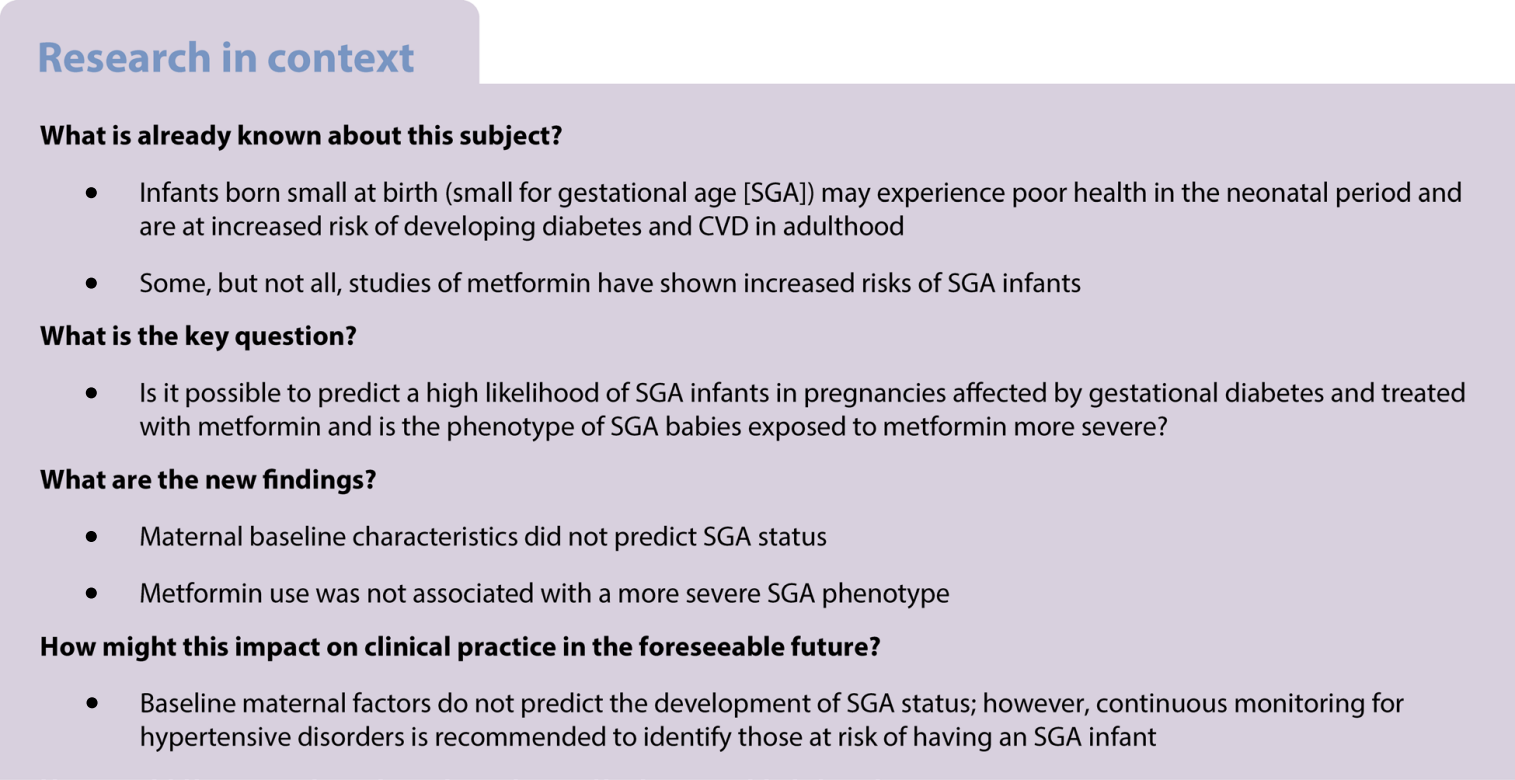


## Introduction

Gestational diabetes mellitus (GDM) is a significant global health problem, affecting 2.93 million pregnancies per year worldwide [1]. Improved glycaemic management nutritional therapy and exercise followed by insulin if necessary [2]) is associated with improved perinatal outcomes [3, 4]. To improve GDM pregnancy outcomes and identify alternative treatments, the EMERGE (effectiveness of early metformin in addition to usual care in the reduction of gestational diabetes effects) trial randomised women with GDM to receive early metformin or placebo in addition to usual lifestyle interventions [5, 6]. The primary outcome was a composite of insulin initiation or a fasting laboratory blood glucose level of ≥5.1 mmol/l at week 32 or week 38 of gestation. A total of 535 pregnancies were randomised, 268 to metformin and 267 to placebo. Participants demonstrated ≥80% adherence to study medications at all trial visits. Participants were randomised before 28+6 weeks’ gestation and mean gestational age at randomisation was 25.5 weeks. There was no statistically significant difference between groups in the primary composite outcome. However, EMERGE reported several benefits of metformin: (1) lower maternal weight gain; (2) lower maternal insulin use; and (3) improved maternal glycaemic management. Infants of metformin-exposed mothers had a lower birthweight and lower rates of large for gestational age infants or macrosomia (>4000 g) and showed no increase in rates of preterm birth or perinatal morbidity. In total, 5.7% of infants in the metformin arm were SGA compared with 2.7% in the placebo arm, resulting in an RR of 2.14 (95% CI 0.89, 5.17), with no significant difference in the proportion of SGA infants by metformin exposure (*p*=0.13).

Metformin may affect protein synthesis, thereby impacting fetal nutrition [7] and having the potential to contribute to small for gestational age (SGA) status. Infants born SGA [8] are reported to have higher infant mortality rates, adverse perinatal morbidity (including neurodevelopmental and cognitive deficiencies [9]) and a higher risk of diabetes and CVD in adulthood [10]. Importantly, infants born SGA to mothers with GDM have a worse prognosis than those born to mothers with normal glucose tolerance [11].

This study aimed to explore predictors of SGA infants using data from the EMERGE trial cohort and to identify risk factors for pregnancies with high odds of SGA infants in which avoidance of metformin may be appropriate. We also sought to explore the effects of maternal metformin exposure on neonatal outcomes in SGA infants.

## Methods

### Study design

This was a prespecified secondary analysis of EMERGE, whose design and primary outcomes have been reported previously [5, 6]. In brief, EMERGE randomised women with GDM (using WHO 2013 criteria [12]) and a live singleton fetus (<28+5 weeks’ gestation at enrolment) to receive metformin (2500 mg/day in two doses) or matched placebo. Race and ethnicity were self-reported and the population was generally representative of the overall population of Ireland, with >70% of respondents self-reporting white or European descent and 18% receiving medical care or state-aided medical funding. In this analysis we included participants with a live-born infant and known infant weight and gestational age at delivery. Ethical approval was obtained from Galway University Hospital and written informed consent was obtained from all participants.

### Outcomes

SGA was defined as infant birthweight <10th percentile for gestational age and infant sex using standard growth curves [13]. Other outcomes included gestational age at delivery, preterm birth (<37 completed weeks’ gestation), birthweight, abdominal head and arm circumference, crown heel length, need for neonatal unit (NNU) care, respiratory distress requiring support, jaundice requiring phototherapy, Apgar (appearance, pulse, grimace, activity and respiration) score <7 at 5 min, neonatal hypoglycaemia (<2.6 mmol/l), pre-eclampsia, gestational hypertension according to the American College of Obstetrics and Gynecology [14] and delivery by Caesarean section.

### Statistical analysis

Baseline maternal characteristics and pregnancy outcomes were compared by SGA status and, within SGA pregnancies, by metformin exposure. Means and SDs, medians and IQRs, and counts and percentages were used, as appropriate. Comparisons were completed using *t* tests, Wilcoxon rank sum tests, χ^2^ tests or Fisher’s exact tests, as appropriate. Logistic regression was used to investigate the following prespecified baseline predictors of SGA status (because of small numbers of SGA infants): pre-pregnancy BMI, known maternal hypertension, BP recorded at randomisation, smoking status, employment status and HbA_1c_.

## Results

We included 98% (*n*=522) of EMERGE pregnancies in this secondary analysis (261 in each arm). In total, 4.20% (*n*=22) of infants were born SGA (5.70% [*n*=15] of the metformin group; 2.70% [*n*=7] of the placebo group; *p*=0.13). Maternal baseline characteristics were similar between mothers with and mothers without an SGA infant (Table 1). There was no statistically significant difference in the maternal requirement for insulin between women with an SGA infant and women without an SGA infant (27.30% [*n*=6] for SGA vs 45.00% [*n*=225] for non-SGA infants; *p*=0.15) (Table 2). In addition, there were no differences in history of prior hypertension or pre-eclampsia between women with and without an SGA infant, and BP was similar in the two groups at randomisation (Table 1).

**Table 1:** Baseline characteristics of EMERGE participants by SGA infant and metformin status

Characteristic	EMERGE trial overall			SGA infants only		
	SGA infants (*N*=22)	No SGA infants (*N*=500)	*p* value	Metformin (*N*=15)	Placebo (*N*=7)	*p* value
Age (years), mean (SD)	33.7 (4.5)	34.4 (4.8)	0.48	33.20(4.3)	34.7 (5.0)	0.51
Race and ethnicity						
White	72.7 (16)	83.4 (417)	0.31	80.0 (12)	57.1 (4)	0.33
Black/Asian/Irish Traveller	27.3 (6)	16.6 (83)		20.0 (3)	42.9 (3)	
Medical card^a^	18.2 (4)	23.6 (118)	0.74	26.7 (4)	0	1.00
Unemployed	4.5 (1)	8.8 (44)	0.76	33.3 (5)	0	1.00
Education						
Tertiary	59.1 (13)	64.4 (322)	0.85	53.3 (8)	71.4 (5)	0.82
Secondary	31.8 (7)	28.8 (144)		33.3 (5)	28.6 (2)	
Primary	9.1 (2)	6.8 (34)		13.3 (2)	0	
Smoking status						
Never	50.0 (11)	51.4 (257)	0.88	53.3 (8)	42.9 (3)	1.00
Former	40.9 (9)	42.2 (211)		40.0 (6)	42.9 (3)	
Current	9.1 (2)	6.4 (32)		6.7 (1)	14.3 (1)	
BMI (kg/m^2^), median (IQR)	29.6 (28.0–38.2)	31.8 (24.9–38.7)	0.22	30.4 (27.0–36.5)	29.5 (27.5–30.0)	0.78
BMI						
≥30 kg/m^2^	45.5 (10)	61.0 (305)	0.22	53.3 (8)	28.6 (2)	0.38
<30 kg/m^2^	54.5 (12)	39.0 (195)		46.7 (7)	71.4 (5)	
Systolic BP (mmHg), mean (SD)^b^	115.5 (10.6)	114.7 (9.3)	0.71	115.6 (9.8)	115.4 (13)	0.98
Diastolic BP (mmHg), mean (SD)^b^	71.8 (9.9)	68.7 (8.4)	0.16	69.0 (8.9)	77.9 (10)	0.07
Gestational age (weeks), mean (SD)^b^	26.2 (3.4)	25.4 (4.2)	0.27	25.9 (4)	27.1 (1)	0.28
HbA_1c_ (mmol/mol), mean (SD)	32.5 (3.6)	33.0 (3.5)	0.54	32.2 (4.0)	33.0 (2.8)	0.61
HbA_1c_ (%), mean (SD)	5.1 (0.3)	5.2 (0.3)		5.1 (0.4)	5.2 (0.30)	
Prior pre-eclampsia	18.2 (4)	8.6 (43)	0.25	13.3 (2)	28.6 (2)	0.56
Prior GDM	9.1 (2)	24.6 (123)	0.16	13.3 (2)	0.0	1.0
Prior macrosomia	9.1 (2)	19.6 (98)	0.34	13.3 (2)	0.0	1.0
Prior hypertension	4.5 (1)	3.0 (15)	1.00	0	14.3 (1)	1.0

Data are % (*n*) unless indicated otherwise

^a^Free medical care at point of need (means tested)

^b^At randomisation

**Table 2:** Neonatal and maternal outcomes of EMERGE pregnancies by SGA infant status

Characteristic	SGA infants (*N*=22)	No SGA infants (*N*=500)	*p* value
Gestational age at birth (weeks)	38.8 (1.4)	39.1 (1.6)	0.36
Preterm birth (<37 weeks), % (*n*)	18.2 (4)	7.4 (37)	0.15
Birthweight (g), mean (SD)	2555.9 (312)	3489 (493)	<0.001
Birthweight <2500 g, % (*n*)	40.9 (9)	3.2 (16)	0.001
Head circumference (cm)	33 (1.8)	34.8 (1.7)	0.001
Crown heel length (cm)	47.2 (2.6)	51.5 (3.1)	0.001
Abdominal circumference (cm)	29.3 (0.7)	33.5 (2.5)	0.001
Upper arm circumference (cm)	9.3 (0.8)	11.1 (1)	0.001
Need for NNU, % (*n*)	36.4 (8)	13.2 (66)	0.006
Days in NNU, median (IQR)	3 (0–52)	3 (0–50)	1.0
Caesarean section delivery, % (*n*)	50.0 (11)	41.4 (207)	0.56
Respiratory distress syndrome, % (*n*)	31.8 (7)	13.6 (68)	0.041
Neonatal hypoglycaemia, % (*n*)	9.1 (2)	11.4 (57)	1.0
Neonatal jaundice, % (*n*)	0	0.4 (2)	1.0
Apgar score <7 at 5 min, % (*n*)	0	0.4 (2)	1.0
Major congenital malformation, % (*n*)	4.5 (1)	3.2 (16)	1.0
Maternal HbA_1c_ at week 36 (mmol/mol)	33.7 (3.4)	34.4 (3.9)	0.47
Maternal HbA_1c_ at week 36 (%)	5.2 (0.3)	5.3 (0.4)	0.47
Maternal fasting glucose week 32 (mmol/l)	4.7 (0.5)	4.9 (0.5)	0.03
Maternal fasting glucose week 38 (mmol/l)	4.3 (0.3)	4.6 (0.5)	0.006
Maternal postprandial glucose week 32 (mmol/l)	5.3 (0.4)	5.5 (0.4)	0.55
Maternal postprandial glucose week 38 (mmol/l)	5.4 (0.5)	5.4 (0.5)	0.88
Maternal insulin requirement, % (*n*)	27.3 (6)	45.0 (225)	0.15
Units insulin/kg week 36, median (IQR)	0.1 (0–1.9)	1.0 (0.9–2.1)	0.44
Maternal weight gain^a^ (kg)	−0.1 (3.5)	1.5 (3.4)	0.16
Pre-eclampsia, % (*n*)	18.2 (4)	2.0 (10)	0.001
Gestational hypertension, % (*n*)	22.7 (5)	5.4 (27)	0.005

Data are mean (SD) unless indicated otherwise

^a^From randomisation to delivery

Similarly, there were no differences in maternal baseline characteristics among SGA pregnancies by metformin exposure (Table 1). Among SGA pregnancies, there was no significant difference in the maternal requirement for insulin by metformin exposure (26.70% [*n*=4] in the metformin group vs 28.60% [*n*=2] in the placebo group; *p*=1.00) (Table 3). On multivariable-adjusted logistic regression, no baseline maternal variables were associated with SGA infants (see electronic supplementary material [ESM] Tables 1 and 2).

**Table 3:** Neonatal and maternal outcomes of SGA pregnancies by metformin status

Characteristic	Metformin (*N*=15)	Placebo (*N*=7)	*p* value
Gestational age at birth (weeks)	38.7 (1.4)	39.1 (1.4)	0.51
Preterm birth (<37 weeks), % (*n*)	20.0 (3.0)	14.3 (1.0)	1.00
Birthweight (g)	2529.3 (312.1)	2612.9 (328.6)	0.88
Birthweight <2500 g, % (*n*)	40.0 (6.0)	42.9 (3.0)	1.00
Head circumference (cm)	32.9 (1.8)	33.2 (2.1)	0.77
Crown heel length (cm)	46.6 (2.7)	48.3 (3.1)	0.17
Abdominal circumference (cm)	29.3 (0.8)	29.2 (0.4)	0.88
Upper arm circumference (cm)	9.3 (0.9)	9.2 (1.1)	0.98
Need for NNU, % (*n*)	13.3 (2.0)	42.9 (3.0)	0.27
Days in NNU, median (IQR)	3.0 (0–52)	3.0 (0–50)	1.00
Caesarean section delivery, % (*n*)	50.0 (11.0)	28.6 (2)	0.25
Respiratory distress syndrome, % (*n*)	26.7 (4.0)	42.9 (3.0)	0.79
Neonatal hypoglycaemia, % (*n*)	6.7 (1.0)	14.3 (1.0)	1.00
Neonatal jaundice, % (*n*)	60.0 (9.0)	28.6 (2.0)	0.36
Apgar score <7 at 5 min, % (*n*)	0	0	1.00
Major congenital malformation, % (*n*)	6.7 (1.0)	0	1.00
Maternal HbA_1c_ week 36 (mmol/mol)	33.4 (3.6)	34.2 (3.3)	0.71
Maternal HbA_1c_ week 36 (%)	5.2 (0.3)	5.3 (0.3)	0.71
Maternal fasting glucose week 32 (mmol/l)	4.7 (0.4)	4.7 (0.5)	0.80
Maternal fasting glucose week 38 (mmol/l)	4.3 (0.4)	4.3 (0.4)	0.97
Maternal postprandial glucose week 32 (mmol/l)	5.2 (0.3)	5.5 (0.5)	0.32
Maternal postprandial glucose week 38 (mmol/l)	5.2 (0.4)	5.6 (0.6)	0.22
Maternal insulin requirement, % (*n*)	26.7 (4.0)	28.6 (2.0)	1.00
Maternal weight gain^a^	0.1 (3.8)	−1.0 (1.4)	0.51
Pre-eclampsia, % (*n*)	26.7 (4.0)	0	0.36
Gestational hypertension, % (*n*)	20.0 (3.0)	28.6 (2.0)	1.0

Data are mean (SD) unless indicated otherwise

^a^From randomisation to delivery

### Pregnancy outcomes

There were no significant differences in gestational age at delivery or number of preterm births by SGA status (Table 2). However, infants who were SGA had lower birthweights and lower anthropometric measurements. They were also more likely to require NNU care and have respiratory distress syndrome, but were not more likely to have neonatal hypoglycaemia, jaundice or major congenital malformations.

Mothers with SGA pregnancies were more likely to have lower fasting (but not postprandial) glucose levels at gestational weeks 32 and 38 and were more likely to develop pre-eclampsia or gestational hypertension (Table 2). Unadjusted logistic regression showed reduced odds of having an SGA infant per 0.1 mmol/l increase in fasting glucose at week 32 (OR 0.90, 95% CI 0.81, 0.99) and week 38 (OR 0.86, 95% CI 0.75, 0.97); after adjustment for both pre-eclampsia and gestational hypertension, statistical significance was lost. Unadjusted logistic regression showed increased odds of having an SGA infant with pre-eclampsia (OR 10.8, 95% CI 2.75, 35.7) and gestational hypertension (OR 5.09, 95% CI 1.58, 14.0) during pregnancy (ESM Table 2); after mutual adjustment, only pre-eclampsia remained significantly associated with having an SGA infant (OR 6.20, 95% CI 1.29, 25.9) (ESM Table 3).

### SGA pregnancy outcomes

Among SGA pregnancies, there were no significant differences in gestational age at delivery, number of preterm births, birthweight, anthropometric measurements, need for NNU care and rates of respiratory distress syndrome, neonatal hypoglycaemia, jaundice and major congenital malformations by metformin exposure (Table 3). Similarly, there were no significant differences in maternal glucose measurements, HbA_1c_, insulin requirements or weight gain by metformin exposure (Table 3). Logistic regression was not performed because of the small number in each group.

## Discussion

In this secondary analysis of the EMERGE trial, the SGA rate was within population norms, with no statistically significant difference by metformin exposure. Our SGA rate (6.2%) was similar to that reported in the MiG trial (8.5%), which also investigated treatment with 2500 mg/day metformin [15]. Notably, the SGA rate was lower than that in the MiTy trial (11.8%), which included women with type 2 diabetes with/without complications, including nephropathy, and investigated a lower dose of metformin (2000 mg/day) [16]. However, notably, renal impairment or nephropathy was an exclusion criterion in the EMERGE study. Participants in the MiTy study also had a mean BMI of 35 kg/m^2^ compared with a mean BMI of 30 kg/m^2^ for the EMERGE participants.

No baseline maternal factors were associated with an increased odds of having an SGA infant (univariate- or multivariable-adjusted analysis). As expected, SGA infant anthropometric measurements were lower than non-SGA infant measurements. Importantly, there were no statistically significant differences in neonatal outcomes by metformin exposure among SGA infants; however, some statistically non-significant differences may have clinical importance (e.g. lower birthweight and higher rate of jaundice in metformin-exposed individuals).

Maternal glycaemic management and insulin requirements were similar between those who did and those who did not have an SGA infant and were not associated with having an SGA infant. Pre-eclampsia and gestational hypertension were significantly associated with SGA infants on univariate analysis, and pre-eclampsia remained so after multivariable adjustment. Although the overall number of SGA infants was small, we found that metformin exposure did not increase the risk of pre-eclampsia and gestational hypertension in the SGA cohort. Taken together, these data suggest that hypertensive disorders of pregnancy may have a greater impact on SGA status in GDM pregnancies, with little impact of metformin.

In the EMERGE trial, metformin was introduced early following a GDM diagnosis, with participants randomised at a median of gestational week 27 and a median duration of treatment of 12 weeks, with no difference between the randomised groups. This differs from current routine practice in which metformin is not introduced until lifestyle treatment fails. Based on this secondary analysis, one would not expect an increase in SGA rates or a more severe SGA phenotype if metformin was introduced earlier in routine clinical practice.

This secondary analysis has several strengths. Data were derived from the EMERGE trial, a large, double-blind randomised trial comparing metformin with placebo in women with GDM, which had a low attrition rate and a high rate of adherence to treatment; in total, 98% of EMERGE pregnancies were included in this secondary analysis. This rigorous methodological approach allowed for a more focused examination of the specific research question while leveraging the high-quality data collected within a well-designed clinical trial framework. Furthermore, by carefully defining our inclusion criteria and using multivariable-adjusted regression analyses, we sought to minimise potential confounding factors and enhance the validity of our findings. This study also has limitations. We acknowledge that it may have been underpowered to detect associations; however, underpowered studies can still provide valuable insights and contribute to the overall body of knowledge in a given field. Although larger studies may be needed to confirm the results reported here, our study provides a valuable starting point for future research and highlights the need for continued investigation into the risk factors for SGA infants in the context of GDM and metformin exposure.

In conclusion, this secondary analysis of the EMERGE trial, although limited by the small sample size, found similar rates of SGA infants to previous trials, no significant increase in the odds of having an SGA infant with metformin and no statistically significant difference in maternal characteristics between those who did and those who did not have an SGA infant. The presence of hypertensive disorders of pregnancy was strongly associated with having an SGA infant, independent of metformin exposure.

## Supplementary Information

Below is the link to the electronic supplementary material.

ESM Tables (PDF 165 KB)

## Data Availability

Data are not available as ongoing and long-term follow-up is underway.

## Abbreviations

Apgar: Appearance, pulse, grimace, activity and respiration
GDM: Gestational diabetes mellitus
NNU: Neonatal unit
SGA: Small for gestational age
