# Supplementary material for: Metformin and small for gestational age babies: findings of a randomised placebo-controlled clinical trial of metformin in gestational diabetes (EMERGE)
Source: Diabetologia. 2024 Aug 31;67(12):2660–6. doi: 10.1007/s00125-024-06252-y (PMC11604746; doi:10.1007/s00125-024-06252-y)
Supplement: Supplementary file 1 — ESM Tables (PDF 165 KB) [file 125_2024_6252_MOESM1_ESM.pdf]

ESM Table 1- Logistic regression for prediction of SGA

Characteristic	OR[1]	95% CI[1]	p-value
Group			
Metformin	—	—	
Placebo	0.50	0.17, 1.34	0.2
Age	0.98	0.87, 1.10	0.7
BMI at screening	0.95	0.85, 1.04	0.3
Gestational Age	0.94	0.74, 1.23	0.6
Race			
Caucasian	—	—	
Other	3.02	0.79, 11.2	0.10
Educational level			
Primary Only	—	—	
Secondary Only	0.65	0.11, 5.39	0.7
Tertiary education	0.50	0.08, 4.19	0.5
Medical card holder			
No	—	—	
Yes	1.12	0.26, 4.24	0.9
Private insurance			
No	—	—	
Yes	1.51	0.49, 4.89	0.5
Employment status			
Other	—	—	
Unemployed	0.50	0.02, 3.48	0.5
Smoking status			
Current	—	—	
Former	0.33	0.06, 2.74	0.2
Never	0.27	0.04, 2.48	0.2
Hypertension			
No	—	—	

Characteristic	OR[1]	95% CI[1]	p-value
Yes	1.35	0.06, 11.4	0.8
Nulliparous			
No	—	—	
Yes	3.21	0.86, 14.3	0.10
Macrosomia			
No	—	—	
Yes	0.67	0.08, 3.52	0.7
Caesarean delivery			
No	—	—	
Yes	1.34	0.31, 5.49	0.7
Miscarriage			
No	—	—	
Yes	1.66	0.59, 4.72	0.3
Previous GDM			
No	—	—	
Yes	0.08	0.00, 1.19	0.12
Pre-eclampsia			
No	—	—	
Yes	3.39	0.63, 15.3	0.12
PP haemorrhage			
No	—	—	
Yes	2.01	0.26, 11.1	0.4
Polyhydraminos			
No	—	—	
Yes	3.20	0.14, 31.4	0.4
Ante-partum haemorrhage			
No	—	—	
Yes	3.11	0.39, 17.2	0.2
SBP	0.99	0.92, 1.05	0.7

**Characteristic**	**OR**[1]	**95% CI**[1]	**p-value**
DBP	1.06	0.99, 1.14	0.13
Glucose at 0mins	0.56	0.17, 1.74	0.3
Glucose at 60mins	1.02	0.76, 1.38	>0.9
Glucose at 120mins	0.99	0.69, 1.42	>0.9
HbA1c	0.99	0.83, 1.18	>0.9

[1]OR = Odds Ratio, CI = Confidence Interval, BMI = Body Mass Index, PP haemorrhage = Post-partum haemorrhage, SBP= Systolic Blood Pressure, DBP= Diastolic Blood Pressure

ESM Table 2 - Impact of hypertensive disorders of pregnancy on SGA risk

Characteristic	OR[1]	95% CI[1]	p-value
Pre-eclampsia			
No	—	—	
Yes	10.8	2.75, 35.7	<0.001
Gestational hypertension			
No	—	—	
Yes	5.09	1.58, 14.0	0.003

[1]OR = Odds Ratio, CI = Confidence Interval

ESM Table 3 – Impact of hypertensive disorders of pregnancy on SGA risk after mutual adjustment

Characteristic	exp(Beta)	95% CI[1]	p-value
Pre-eclampsia			
No	—	—	
Yes	6.20	1.29, 25.9	0.015
Gestational hypertension			
No	—	—	
Yes	2.72	0.65, 9.06	0.13

[1]CI = Confidence Interval
